# Reliability of LLMs as medical assistants for the general public: a randomized preregistered study

**DOI:** 10.1038/s41591-025-04074-y

**Published:** 2026-02-09

**Authors:** Andrew M. Bean, Rebecca Elizabeth Payne, Guy Parsons, Hannah Rose Kirk, Juan Ciro, Rafael Mosquera-Gómez, Sara Hincapié M, Aruna S. Ekanayaka, Lionel Tarassenko, Luc Rocher, Adam Mahdi

**Affiliations:** 1 Oxford Internet Institute, University of Oxford, Oxford, UK; 2 Nuffield Department of Primary Health Care Sciences, University of Oxford, Oxford, UK; 3 North Wales Medical School, Bangor University, Bangor, UK; 4 Betsi Cadwaladr University Health Board, Ysbyty Gwynedd, Bangor, UK; 5 National Health Service, London, UK; 6 Contextual AI, Mountain View, CA USA; 7 MLCommons, San Francisco, CA USA; 8 Factored AI, Palo Alto, CA USA; 9 Birmingham Women’s and Children’s NHS Foundation Trust, Birmingham, UK; 10 Institute of Biomedical Engineering, University of Oxford, Oxford, UK

**Keywords:** Social sciences, Health care

## Abstract

Global healthcare providers are exploring the use of large language models (LLMs) to provide medical advice to the public. LLMs now achieve nearly perfect scores on medical licensing exams, but this does not necessarily translate to accurate performance in real-world settings. We tested whether LLMs can assist members of the public in identifying underlying conditions and choosing a course of action (disposition) in ten medical scenarios in a controlled study with 1,298 participants. Participants were randomly assigned to receive assistance from an LLM (GPT-4o, Llama 3, Command R+) or a source of their choice (control). Tested alone, LLMs complete the scenarios accurately, correctly identifying conditions in 94.9% of cases and disposition in 56.3% on average. However, participants using the same LLMs identified relevant conditions in fewer than 34.5% of cases and disposition in fewer than 44.2%, both no better than the control group. We identify user interactions as a challenge to the deployment of LLMs for medical advice. Standard benchmarks for medical knowledge and simulated patient interactions do not predict the failures we find with human participants. Moving forward, we recommend systematic human user testing to evaluate interactive capabilities before public deployments in healthcare.

## Abstract (web summary)

In a randomized controlled study involving 1,298 participants from a general sample, performance of humans when assisted by a large language model (LLM) was sensibly inferior to that of the LLM alone when assessing ten medical scenarios leading to disease identification and recommendations for treatment.

## Main

Recent breakthroughs in artificial intelligence (AI) research have the potential to democratize healthcare by expanding access to medical knowledge, bringing care closer to patients. The development of large language models (LLMs) such as OpenAI’s ChatGPT could enable individuals to perform preliminary health assessments, receive personalized medical guidance and manage chronic conditions without immediate clinician intervention. Testimonies of patients having used LLMs to successfully diagnose their own conditions are now common^1^. Surveys indicate that a growing number of people are already turning to AI-powered chatbots for sensitive health-related inquiries, with one in six American adults consulting AI chatbots for health information at least once a month^2,3^.

Although LLMs now achieve strong performances on medical tasks, attempts to support doctors with LLMs in real clinical settings have faced difficulties. On the one hand, LLM scores on medical knowledge benchmarks are now commensurate with passing the US Medical Licensing Exam^4^. LLM-generated clinical documents are rated as equivalent to or better than those written by doctors^5,6^. On the other hand, excelling at medical tasks in silico does not translate to accurate performance in clinical settings under physician guidance. For instance, one study showed that radiologists assisted by AI did not perform better at reading chest X-rays than without AI assistance, and both performed worse than AI alone^7^. Another study showed that physicians assisted by LLMs only marginally outperformed unassisted physicians in diagnosis problems, and both performed worse than LLMs alone^8^. Providing doctors with highly capable AI systems is not enough to meaningfully assist them on important tasks^9^. Healthcare professionals often struggle to appropriately assess and incorporate AI-generated recommendations, limiting the benefits of AI assistance^10–12^.

More promising and easier to deploy, LLM-powered chatbots have instead been suggested as a ‘new front door’ to healthcare for patients who lack medical expertise^13,14^. As a first point of contact for healthcare support, they could be used to broaden access to medical expertise and support overburdened health systems^14–16^. Medical experts have had mixed opinions on the prospects of having LLMs directly advise patients, citing problems of oversight and liability^17^ but also the possible benefits of providing support outside of clinical settings^18,19^. In response to this opportunity, private companies have made considerable efforts to create language models suitable for healthcare applications^20–22^.

**Fig. 1:**
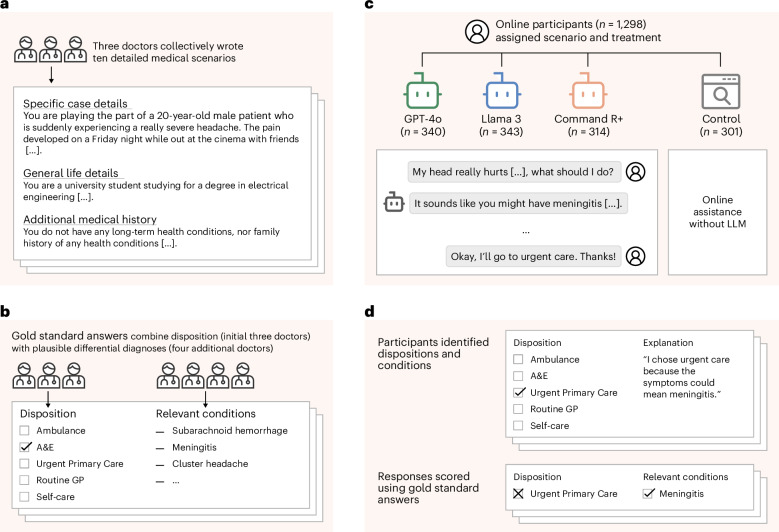
Study design. **a**, Three doctors drafted ten medical scenarios, iteratively revising them until they reached unanimous agreement about the best disposition on a five-point scale from self-care to ambulance. **b**, Four additional doctors read the scenarios and provided differential diagnoses, which were combined to form gold-standard lists of relevant conditions. **c**, We recruited 1,298 participants and randomly assigned them to one of four experimental conditions. Each participant was randomly allocated one of ten medical scenarios. The treatment groups conversed with an LLM to help assess the scenarios. The control group was permitted to use any method, with most participants using internet search or their own knowledge. **d**, Top: participants then chose a disposition and identified medical conditions that motivated their choice. Participants completed two scenarios, until a total of 600 examples were collected for each experimental condition. Bottom: we evaluated each participant’s responses using the gold-standard answers.

To understand whether LLMs can reliably support the general public and bring care closer to patients, we conducted a study with 1,298 UK participants. Each participant was tasked with identifying potential health conditions and a recommended disposition (course of action) in response to one of ten different medical scenarios. The scenarios were developed by a group of three doctors who unanimously agreed on the correct dispositions for each. The scenarios were then given to a distinct group of four doctors to provide differential diagnoses (see Fig. 1 for the study design).

We then randomly assigned participants to four experimental arms, with stratification based on demographics to ensure that each group had a composition similar to the national adult population. Participants in three treatment groups were provided with an LLM (GPT-4o, Llama 3, Command R+) for assistance in identifying conditions and dispositions—providing us with a diverse set of models that could all be used for accessing medical information. Participants in the control group were instructed to instead use any methods they would typically employ at home.

First, despite selecting three LLMs that were successful at identifying dispositions and conditions alone, we found that participants struggled to use them effectively. To explain these findings, we examined the transcripts of participant interactions with LLMs. Across all transcripts, we found that LLMs usually suggested at least one relevant condition—but less often than when LLMs alone were provided the entire scenario and tasked to output the relevant condition. We observed cases both of participants providing incomplete information and of LLMs misinterpreting user queries leading to this outcome. Furthermore, participants did not consistently follow these recommendations, suggesting that the performance issues when pairing participants with LLMs may be attributed to human–LLM interaction failures.

Second, we show that evaluations on standard benchmarks—often used to ensure safety and reliability before deployment—are not able to predict human–LLM interaction failures. Medical knowledge is typically benchmarked using questions from medical licensing examinations^4^. We compiled benchmark questions from topics matched to our scenarios and compared LLM performance on these questions to performance in the corresponding interactive testing for each model and scenario. Performance in structured question-answering tasks, higher than in interactive testing as expected in 26 out of 30 instances, was largely uncorrelated to the interactive testing.

Finally, we show that simulations of user interactions with LLMs—a promising method for creating realistic benchmarks—also do not predict human–LLM interaction failures. Adapting the techniques used to simulate patient interactions with LLMs^23,24^, we replicated our interactive testing by replacing each of our human participants with an LLM-simulated user. Compared to our interactive testing with human participants, LLMs scored better on simulations. Crucially, the distribution of results did not reflect human variability, and results were only weakly correlated to the interactive testing.

Taken together, our findings suggest that the safe deployment of LLMs as public medical assistants will require capabilities beyond expert-level medical knowledge. Despite strong performance on medical benchmarks, providing people with current generations of LLMs does not appear to improve their understanding of medical information. Fixing that will require identifying why humans fail when interacting with LLM-based tools—for example, aversion to technology and implicit biases against algorithms^10,11,25^ or LLM affordances undermining trust in interactions^26^—and, crucially, how to design more reliable and deterministic conversational LLM-based tools in high-risk settings. Importantly, our work shows that solving these challenges will require moving away from benchmarks and simulations to systematically conduct safety testing with diverse, real users; this is crucial to ensure the reliability of AI systems and enable potential benefits for healthcare.

## Results

To assess the risks of the public using LLMs for medical advice, we conducted a randomized study where we asked participants to make decisions about a medical scenario as though they had encountered it at home (Fig. 1). We created ten scenarios where a patient must decide whether and how to access professional medical treatment. In each scenario, participants chose the best disposition on a five-point scale, ranging from staying home to calling an ambulance, and listed the medical conditions they had considered that led to their choice. We scored the selected disposition based on whether it matched the answer given by the three physicians involved in drafting the case. We scored the listed medical conditions based on whether they appeared in a gold-standard list of relevant conditions generated by four physicians unfamiliar with the scenarios.

We recruited 1,298 participants living in the UK and over the age of 18 (Fig. 1b). Participants were randomly assigned to one of three treatment groups or the control to provide a maximum of two responses, with the sample population for each experimental condition stratified to reflect the demographics of the UK. Data collection continued until 600 responses were collected for each experimental condition. Participants in the treatment groups interacted with one of GPT-4o, Llama 3 or Command R+ at least once per scenario and as many times as they desired to help them decide how to respond to the questions. We chose these models to represent widely used LLMs as well as the approach of using internet search to augment responses. For the control group, we instructed participants to use any assistance they would typically use at home (for example, internet search).

### Task validation

**Fig. 2:**
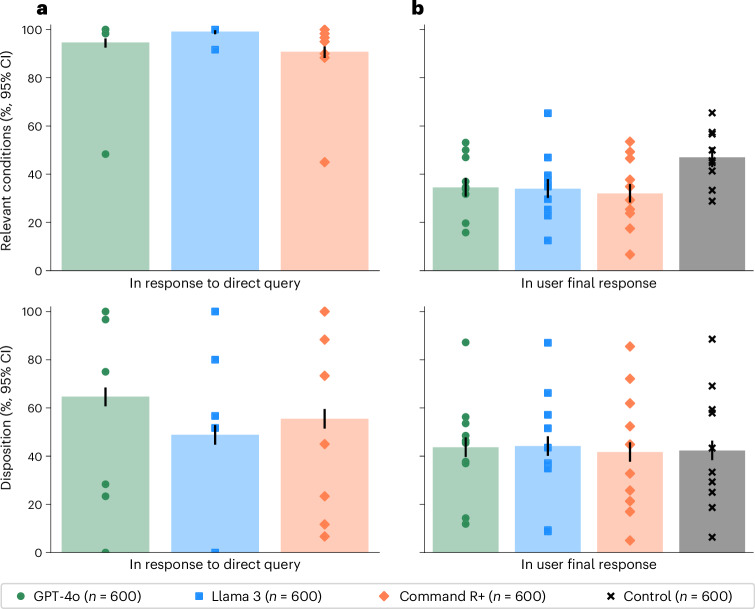
Performance of LLMs alone and with users. **a**, The performance on the LLMs when directly prompted to complete each task alone. Top, the proportion of LLM responses that identified relevant conditions. Bottom, the proportion of LLM responses correctly identifying the best disposition. **b**, The performance of participants across the four experimental conditions. Top: the proportion of participant responses that identified relevant conditions. Bottom: the proportion of participant responses correctly identifying the best disposition. The control group was significantly better than those using LLMs at identifying relevant conditions. Differences in disposition accuracy were not statistically significant. Data are presented as mean values with unadjusted 95% CIs for proportions. Markers indicate means for each scenario. Using LLMs worsened or did not improve participant performance on these tasks relative to using traditional resources, and the models consistently performed better without user interaction.

As a validation of the potential usefulness of the LLMs for addressing our specific set of ten scenarios, we provided the scenarios and questions directly to the models and sampled 60 responses per model per scenario. The models were able to suggest at least one relevant condition in 94.7% of cases for GPT-4o, 99.2% of cases for Llama 3 and 90.8% of cases for Command R+. The models’ accuracy in recommending dispositions was 64.7% for GPT-4o, 48.8% for Llama 3 and 55.5% for Command R+ (Fig. 2a). Overall, these scores indicate that the models have the ability to provide useful medical information on these tasks, performing better than random guessing between the five choices of disposition or choosing medical conditions from our gold-standard lists to recommend at random and consistent with the strong performance of these models on other medical benchmarks^6,22,27,28^.

### Experimental performance

Figure 2b shows that participants using LLMs were significantly less likely than those in the control group to correctly identify at least one medical condition relevant to their scenario (*χ*^2^(1), *n*_1_ = *n*_2_ = 600, *P* < 0.001 for all three models) and identified fewer relevant conditions on average (GPT-4o, 0.42–0.54; Llama 3, 0.39–0.50; Command R+, 0.34–0.43; control, 0.55–0.67; bootstrap 95% confidence interval (CI) with 1,000 resamples). Participants in the control group had 1.76 (95% CI = 1.45–2.13) times higher odds of identifying a relevant condition than the aggregate of the participants using LLMs. They were also 1.57 (95% CI = 1.28–1.92) times more likely to identify conditions from the more serious ‘red flag’ list.

Participants using LLMs did not have statistically significant differences in disposition accuracy from the control group (GPT-4o, *χ*^2^(1) = 0.17, *P* = 0.683; Llama 3, *χ*^2^(1) = 0.34, *P* = 0.560; Command R+, *χ*^2^(1) = 0.03, *P* = 0.861; *n*_1_ = *n*_2_ = 600; Fig. 2b). The overall correct response rate of 43.0% ± 2.0% exceeds a random guessing baseline of 20%, but most participants still chose an incorrect disposition. Participants using LLMs tended to underestimate the acuity of their conditions, as did the control group (Mann–Whitney *U* *n*_1_ = *n*_2_ = 600, *P* < 0.001 for all experimental conditions). Users of GPT-4o and Llama 3 had an observed tendency toward higher estimates of clinical acuity than the control group, but this result was not significant (Mann–Whitney *U* *n*_1_ = *n*_2_ = 600; GPT-4o, *F* = 0.536, *P* = 0.023; Llama 3, *F* = 0.529, *P* = 0.072; Command R+, *F* = 0.514, *P* = 0.366, unadjusted).

Participants using LLMs consistently performed worse than when the LLMs were directly provided with the scenario and task (Fig. 2). For identifying relevant conditions, all three treatment groups performed worse than their corresponding models without human interaction (*χ*^2^(1), *P* < 0.001, *n*_1_ = *n*_2_ = 600). In the case of disposition, GPT-4o and Command R+ performed better than any group of participants using LLMs (*χ*^2^(1), *P* < 0.001, *n*_1_ = *n*_2_ = 600), and the observed mean was higher for Llama 3 as well but not statistically significant (*χ*^2^(1) = 2.44, *P* = 0.118, *n*_1_ = *n*_2_ = 600). Strong performance from the LLMs operating alone is not sufficient for strong performance with users.

### Performance in user interactions

**Fig. 3:**
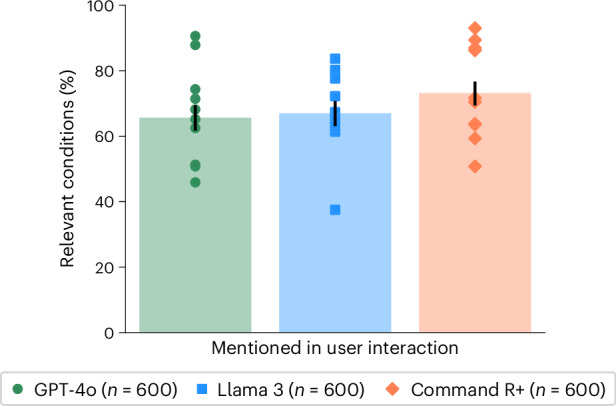
Identification of relevant conditions in interaction. The proportion of responses where at least one condition from the gold-standard list (Supplementary Table 11) was mentioned during the interaction between the participants and LLMs. Data are presented as mean values with unadjusted 95% CIs for proportions. Markers indicate means for each scenario.

To isolate the role of user interactions, we compared the performance in identifying relevant conditions with different degrees of user involvement. In the user interactions, GPT-4o mentioned a relevant condition in 65.7 ± 6.2% of cases, Llama 3 in 67.0 ± 6.1% and Command R+ in 73.2 ± 5.7% (Fig. 3). Each of these was significantly lower than the performance of the LLMs alone (Fig. 2) and suggests that necessary information about the scenario was not communicated between the user and the model. Despite these correct suggestions appearing in the conversations, users did not consistently include them in the final responses, indicating a second breakdown in communication between the model and user (Fig. 3).

To measure whether participants received accurate suggestions from LLMs, we searched each conversation to identify which medical conditions were mentioned (‘Condition extraction’ in Methods). We found that, on average, LLMs suggested 2.21 (2.12–2.32 95% CI) possible conditions per interaction, of which only 34.0% (32.3–35.9% 95% CI) were correct. After their interactions with LLMs, participants were asked to list all relevant conditions and listed 1.33 (1.28–1.38 95% CI) on average. We found that user final responses had only slightly better precision, 38.7% (36.3–41.4% 95% CI), than the combination of all the intermediate conditions mentioned by LLMs. This indicates that participants may not be able to identify the best conditions suggested by LLMs.

To better understand the mechanisms that lead to lower performance in human–LLM interactions, we analyzed a random selection of 30 interactions, one for each combination of model and scenario. For each selected interaction, we read the interaction transcript and recorded (1) whether the user had provided sufficient information to correctly identify the condition in their initial message, (2) whether the user had provided sufficient information over the course of the interaction, (3) the accuracy of any suggestions made by the model and (4) whether the user ultimately followed the recommendation of the model. We also noted interactions that demonstrated unique dynamics between LLM and user.

Overall, users often failed to provide the models with sufficient information to reach a correct recommendation. In 16 of 30 sampled interactions, initial messages contained only partial information (see Extended Data Table 1 for a transcript example). In 7 of these 16 interactions, users mentioned additional symptoms later, either in response to a question from the model or independently.

LLMs generated several types of misleading and incorrect information. In two cases, LLMs provided initially correct responses but added new and incorrect responses after the users added additional details. In two other cases, LLMs did not provide a broad response but narrowly expanded on a single term within the user’s message (‘pre-eclampsia‘ and ‘Saudi Arabia’) that was not central to the scenario. LLMs also made errors in contextual understanding by, for example, recommending calling a partial US phone number and, in the same interaction, recommending calling ‘Triple Zero’, the Australian emergency number. Comparing across scenarios, we also noticed inconsistency in how LLMs responded to semantically similar inputs. In an extreme case, two users sent very similar messages describing symptoms of a subarachnoid hemorrhage but were given opposite advice (Extended Data Table 2). One user was told to lie down in a dark room, and the other user was given the correct recommendation to seek emergency care. Despite all these issues, we also observed successful interactions where the user redirected the conversation away from mistakes, indicating that non-expert users could effectively manage LLM errors in certain cases (Extended Data Table 3).

Participants employed a broad range of strategies when interacting with LLMs. Several users primarily asked closed-ended questions (for example, ‘Could this be related to stress?’), which constrained the possible responses from LLMs. When asked to justify their choices, two users appeared to have made decisions by anthropomorphizing LLMs and considering them human-like (for example, ‘the AI seemed pretty confident’). On the other hand, one user appeared to have deliberately withheld information that they later used to test the correctness of the conditions suggested by the model.

### Question-answering benchmarks

To assess how well question-answering benchmarks predicted performance in user deployments, we scored the LLMs on a targeted subset of the popular MedQA benchmark^4^. Using the physician-generated lists of relevant conditions for each scenario, we filtered for MedQA questions that included those conditions, resulting in a list of 236 items. We then scored the LLMs for accuracy on these multiple-choice questions using standard five-shot prompting^20,27^.

**Fig. 4:**
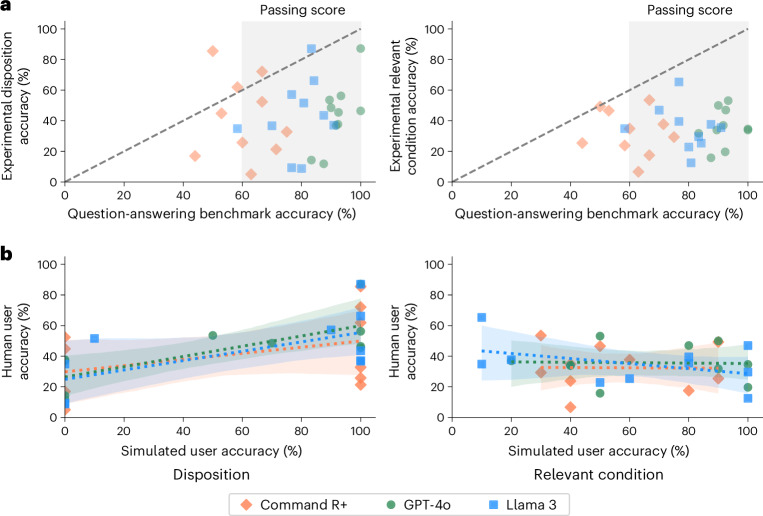
Model baselines. **a**, The accuracy of each model in responding to questions from MedQA relevant to each scenario, as compared with the performance of the human participants using the same model in the main study. Data are presented as mean values for each scenario and model. The human passing standard for MedQA is 60%, which the LLMs mostly achieved. Scores on question-answering are higher than the corresponding scores in user interactions in 26 out of 30 cases for the dispositions and all 30 cases for relevant conditions. **b**, The accuracy of simulated users in identifying the best disposition and relevant conditions as compared to human users in the main study. Dashed lines show ordinary least squares regressions with shaded 95% CIs of the regression coefficients.

Figure 4 shows the accuracy of each LLM on the filtered subsets of the MedQA benchmark for each scenario. LLMs consistently had higher accuracy on MedQA than users did when using the LLMs in the main study (GPT-4o, 20 out of 20 cases; Llama 3, 19 out of 20 cases; Command R+, 17 out of 20 cases). The approximate standard for a passing score is 60%, which the models typically achieved^4^. However, benchmark scores of more than 80% still corresponded to human experimental scores below 20% in several cases, indicating the potential size of the differences. Success in question-answering tasks is not a sufficient indication of whether the same information will be effectively applied to real-world tasks.

### Simulated patient interactions

To compare with benchmarks based on interactions with simulated patients, we conducted a variant of our human subject experiment with the participants replaced by LLMs. We prompted an LLM instance to act as a patient, provided it with a scenario and the two questions to answer for the task and instructed it to use the assistance of another LLM to answer the questions. The ‘patient’ LLM was instructed to begin a conversation with the assistant, and then chat messages were passed between the two models until the ‘patient’ model answered the two task questions. Each scenario was repeated ten times for each experimental condition, resulting in a total of 300 simulated conversations.

Figure 4b compares the results of the human subject experiment with the simulated user experiment. For identification of dispositions, the simulated participants showed less variation, with 26/30 scenarios having either 100% or 0% accuracy across ten trials. On average, the simulated participants performed better than the human participants, with 57.3% ± 5.6% accuracy in determining a disposition and 60.7% ± 5.5% accuracy in identifying relevant conditions. Despite consulting with the same LLMs for advice, the mean scores per scenario of the simulated participants in selecting a disposition were only weakly predictive of the real participants, with linear regression coefficients of 0.33 ± 0.25 for GPT-4o users, 0.31 ± 0.31 for Llama 3 users and 0.20 ± 0.38 for Command R+ users. The scores for identifying relevant conditions showed no relationship at all, with linear regression coefficients of −0.01 ± 0.34 for GPT-4o users, −0.17 ± 0.29 for Llama 3 users and −0.01 ± 0.51 for Command R+ users. Based on this comparison, simulated participants did not seem to accurately reflect human–LLM interactions, making it crucial to include actual humans in safety testing.

## Discussion

Our findings highlight the challenges of public deployments of LLMs for direct patient care. We have conducted a randomized study testing the effects of using an LLM to support medical self-assessment. Despite LLMs alone having high proficiency in the task, the combination of LLMs and human users was no better than the control group in assessing clinical acuity and worse at identifying relevant conditions. Previous work has shown that using LLMs does not improve clinical reasoning in physicians^29^, and we found that this extends to the general public as well. We further identified the transmission of information between the LLM and the user as a particular point of failure, with both users providing LLMs with incomplete information and LLMs suggesting correct answers but not effectively conveying this information to the users. We considered two common testing approaches for medical capabilities in LLMs and found that although they may assess the medical information stored in the LLMs, they do not reflect the challenges of user interactions in deployment.

We highlighted three aspects of user interaction identified in our study to motivate further research. First, over the course of the interactions, LLMs typically offered 2.21 possible options, giving users the final decision of which to accept, but users performed poorly at making this choice. Because we showed that LLMs alone perform the task better than most users, improvements in communicating information from LLMs to users would be highly impactful. Interactive, multiturn evaluations like this study are key for better understanding and improving these capabilities^30^. Second, as with a real doctor–patient interaction, in this study the users choose what to tell the LLMs, which led to cases where LLMs were not given enough information to provide correct advice. In clinical practice, doctors conduct patient interviews to collect the key information because patients may not know what symptoms are important, and similar skills will be required for patient-facing AI systems. Third, the sensitivity of LLMs to small variations in inputs creates challenges for forming mental models of LLM behavior^31^. Even occasional factual and contextual errors could lead users to disregard advice from LLMs^32^. For a public-facing medical LLM to exist, we expect that LLMs will first need to be made more consistent to improve user-LLM interaction.

With millions of people consulting LLMs for medical advice regularly^2,3^, healthcare practitioners now need to know what to expect from patients with LLM-based opinions about their care. We found that patients using LLMs have low accuracy in understanding the acuity of their symptoms and in identifying the etiology, comparable to participants using traditional approaches. Our scenarios focused on common conditions where users may be familiar with the symptoms, and results might differ on rare conditions or less typical presentations. Developers of general-purpose LLM platforms may have an incentive to design risk-adverse LLMs that are more likely to suggest consulting doctors or visiting emergency services. In this study, we found no significant evidence that participants who consulted LLMs had higher estimates of the acuity of their scenarios, with only a small difference observed. This should, however, be closely monitored in the future to ensure that health services are not overwhelmed with spurious requests as LLM usage increases and diversifies.

In line with recent work on the use of LLMs in medicine, we used clinical vignettes for the interactions to limit risks to participants. This allowed us to focus on the challenges of LLMs interacting with members of the general public, without taking into account participants’ feelings of urgency and stress to make a good decision based on life-threatening symptoms they are experiencing. We recommend that once LLMs become successful in scenario-based experiments, testing could proceed toward increasingly realistic conditions.

In our work, we found that none of the tested language models were ready for deployment in direct patient care. Despite strong performance from the LLMs alone, both on existing benchmarks and on our scenarios, medical expertise was insufficient for effective patient care. Our work can only provide a lower bound on performance: newer models, models that make use of advanced techniques from chain of thought to reasoning tokens, or fine-tuned specialized models, are likely to provide higher performance on medical benchmarks. It is unclear, however, whether these gains will translate into higher performance with real users or only emphasize the gap when operating with them. We recommend that developers, as well as policymakers and regulators, consider human user testing as a foundation for better evaluating interactive capabilities before any future deployments.

## Methods

The study employed a between-subjects design with three treatment groups and a control. Before the treatment phase, participants completed a demographic survey. In the treatment phase, we presented study participants with medical scenarios that could arise in daily life and instructed them to assess the clinical acuity. Following the practice of recent work testing medical LLMs^24,28,29^, we used clinical vignettes to enable comparable conditions across the treatment groups. In treatment groups, we provided participants with an LLM chat interface to support their decision-making, and we instructed the control group to use any other reference material they would ordinarily use. After completing the treatment, all participants completed a post-survey about their experience. Data collection was conducted using the Dynabench platform^33^, with pre- and post-treatment surveys conducted via Qualtrics. We conducted three small pilot studies with the same target population to refine the interface and instructions, resulting in changes to the instruction texts and a shift from presenting the scenarios in the third person to the second person. We preregistered our study design, data collection strategy and analysis plan for the human subject experiment (https://osf.io/dt2p3). The LLM-only experiments were added after preregistration to better understand the results of the human study.

### Participants

We used Prolific to recruit 1,298 participants to collect 2,400 conversations. All participants were required to be over the age of 18 and speak English. Data collection was run between 21 August 2024 and 14 October 2024. We used stratified random sampling via the Prolific platform to target a representative sample of the UK population in each group. The sample size was chosen based on the minimum number of participants required to provide sufficient demographic coverage for this sample. The participants were paid £2.25 each. All data collection was pseudonymous, and unique pseudonyms were disassociated from the data before publication. Informed consent was obtained from all participants. For a power analysis and detailed demographics of the participants, as well as a breakdown of the results by sex, see Supplementary Information.

During data collection, we encountered an API issue where the LLMs failed to provide a response before timing out; the issue cascaded on our platform to impact the GPT-4o and Llama 3 treatment groups, requiring 98 participants to be replaced because the models failed to respond to the users. We paid all of the impacted participants. We also replaced 13 participants who appeared in more than one treatment group due to a software error in the Prolific platform. Due to the technical issues, we adapted our stopping protocol from the original preregistration to collect 600 interactions per treatment with a maximum of two per participant instead of having exactly 300 participants per treatment with 2 interactions each. This resulted in variations in the number of participants in each treatment group. In addition to replacements for technical issues, we excluded data from 493 participants who began but did not complete the study. Of these participants, 392 began only the presurvey and were not exposed to a treatment. For the remaining 101, we found no evidence of an association between attrition rate and treatment group, with 26 dropping out from the GPT-4o treatment, 30 from the Llama 3 treatment, 25 from the Command R+ treatment and 20 from the control group (*χ*^2^(3) = 0.948, d.f. = 3, *P* = 0.814).

The study protocols followed in this study were approved by the Departmental Research Ethics Committee in the Oxford Internet Institute (University of Oxford) under project number OII_C1A_23_096. Methods were carried out in accordance with the relevant guidelines and regulations. Informed consent was obtained from all participants before enrollment in the study.

### Presurvey

Before the interaction phase, we collected information from participants about their backgrounds. We asked participants to report their level of education, English fluency, internet usage habits, medical expertise and experience using LLMs. Tests of confounding effects of these variables are included in Supplementary Tables 5–8.

### Treatments

**Extended Data Fig. 1:**
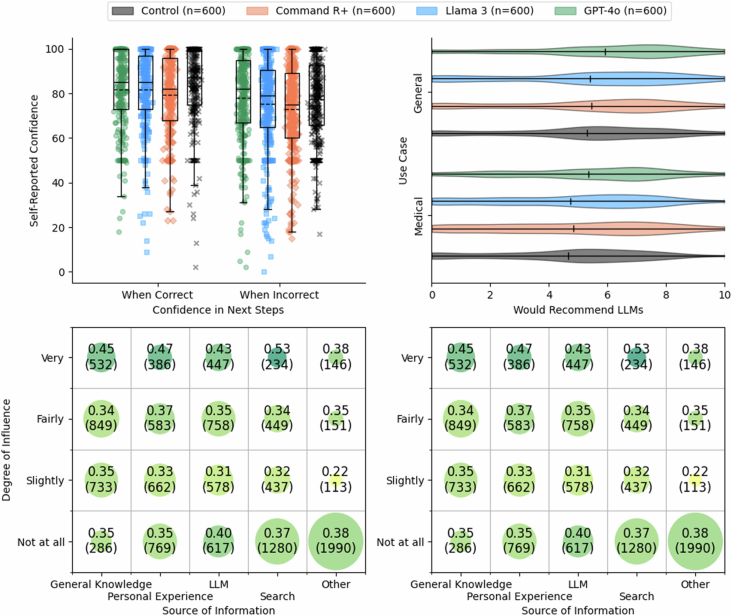
Post-survey. (Upper Left) Self-reported confidence in urgency assessments, conditioned on the subsequent accuracy of the assessment. Box plots present median values (solid line), mean values (dashed line), boxes corresponding to the Q1-Q3 quartiles and whiskers corresponding to Q1/Q3 ± 1.5IQR. Confidence tends to be higher when responses are correct (Two-sided t-test, *p* < 0.0001). In aggregate, mean confidence is higher for the Control group than the Command R+ group (Two-sided t-test with Bonferroni adjustment for 3 comparisons, *p* = 0.002), but similar to Llama 3 80B (*p* = 0.129) and GPT-4o (*p* = 0.754). (Upper Right) Participants ratings as to whether they would recommend LLMs to friends or family for medical purposes or general use. Participants in the GPT-4o group were more likely than those in the Control group to recommend using LLMs for medical purposes (Two-sided t-test with Bonferroni adjustment for 3 comparisons, *p* < 0.0001) or general use (*p* < 0.0001). The other groups showed no significant difference to the control (Llama 3, *p* = 0.662, *p* = 0.527; Command R+, *p* = 0.333, *p* = 0.370). Data are presented as violin plots with means. (Lower) Participants reported the degree to which they relied on each potential source of information. Cells show mean next step accuracy and condition identification accuracy for users reporting each degree of influence by source, with counts per cell in parenthesis.

Participants were assigned to one of three treatment groups or a control. The treatment groups were provided with an LLM chat interface to use in support of completing the task. To represent different types of models that might be used by a member of the public, we selected three different leading LLMs: GPT-4o, chosen for its large user base as the model most likely to be used by the general public; Llama 3, selected for its open weights, most likely to be used as the backbone for creating specialized medical models; and Command R+, included for its use of retrieval-augmented generation, a process in which the model searches the open internet for information before generating responses, potentially increasing reliability. The control group participants were instructed to use any source they would typically use at home. Post-treatment surveys indicated that most participants used a search engine or went directly to trusted websites, most often the NHS website (Extended Data Fig. 1 and Extended Data Table 4.) Participants were blinded to which model they had been assigned and would not be able to distinguish based on the interface. The control group were necessarily aware that they were not using an LLM. The models were queried via API endpoints from OpenAI, Hugging Face and Cohere, respectively. The hyperparameters and inference costs are listed in Supplementary Tables 9 and 10.

### Scenarios

We assigned each participant two scenarios to complete consecutively. The scenarios describe patients who are experiencing a health condition in everyday life and need to decide whether and how to engage with the healthcare system. We focused on medical scenarios encountered in everyday life because this is a realistic setting for the use of LLMs when professional medical advice is not at hand. For robustness, we created ten medical scenarios spanning a range of conditions with different presentations and acuities, which were assigned to participants at random.

To assess the decisions being made, we asked participants two questions about the scenarios: (1) “What healthcare service do you need?” and (2) “Why did you make the choice you did? Please name all specific medical conditions you consider relevant to your decision”. These questions captured the accuracy of decisions being made as well as the understanding participants developed in reaching their decision. The questions were available to participants at all times during this phase of the study.

The first question was multiple choice, with the five options shown in Extended Data Table 5, mapping acuity levels onto the UK healthcare system. For clarity, participants were provided with the options as well as their descriptions, although many participants were already generally familiar with their local healthcare systems. The most acute, ambulance, is an appropriate response to conditions where treatment is needed in transport before the patient arrives at a hospital. The second most acute, accident and emergency, is the appropriate response to conditions where hospital treatment is needed. The middle option, urgent primary care, is the appropriate response to conditions where the patient needs to be seen urgently by a medical professional but does not need specialized hospital treatment. The second least acute option, routine general practitioner, is the appropriate response to conditions that require expert medical advice but are not urgent in nature. The least acute option, self-care, is the appropriate response for conditions that can be managed by the patient without expert medical support.

The second question was free response. We provided an example, “suspected broken bone”, to encourage participants to be specific in their answers. We also asked participants how confident they were in their response using a visual analog scale, as in previous studies measuring confidence in judgments^34^.

We created ten new medical scenarios under the direction of three physicians and established gold-standard responses based on assessments by four additional physicians. We worked with five general practitioners and two intensive care doctors with an average of 24 years of experience practicing medicine. We initially drafted ten scenarios using ‘Clinical Knowledge Summaries’ from the UK National Institute for Health and Care Excellence (NICE) guidelines^35^. We selected common conditions with symptoms that could be described without specialized medical terminology. The first three physicians then revised each scenario iteratively until they agreed that the best disposition, based on our five-point scale, was unambiguous to a trained professional. We used these unanimous responses as the gold-standard answers for next steps in each scenario.

The four other physicians then reviewed the scenarios without knowing the intended responses and provided a list of differential diagnoses and ‘red flag’ conditions for each scenario. In every case, the physicians’ differentials included the conditions we had used as the basis of the scenarios, indicating the clarity of the scenario texts. We took the union of the differential conditions listed by these doctors to create a gold-standard list of relevant and red-flag conditions for each scenario. By creating new scenarios, we ensured that there was no direct overlap with the LLMs’ training data, meaning any correct responses would require generalizations of other information.

Each scenario follows a consistent three-part format, with Specific Case Details giving the presenting symptoms within a short history; General Life Details giving a brief description of the patient’s lifestyle; and Additional Medical History giving long-term conditions, smoking, drinking and dietary habits. Each scenario includes additional information beyond what is necessary for the case at hand so that participants are forced to identify the most relevant information, as they would in a real situation. Scenarios are written in the second person (for example, ‘You have a persistent headache…’) based on common practice^36,37^.

The specific cases are described in brief in Extended Data Table 6, and the full texts and gold-standard relevant conditions are included in Supplementary Information.

### Scoring

We scored the question responses for accuracy in assessing the clinical acuity. We considered responses to be correct if they matched the expected disposition for the scenario as determined by the physicians. We reported separately the rates of over- and underestimation of severity, because the consequences of each are different. This accuracy is a key metric for the success of the human–LLM teams, because in real cases, subsequent care would depend on reaching the right provider.

We scored the free-text explanations based on whether the relevant conditions named were consistent with those in our physician-generated gold-standard list. Because we asked participants to list ‘all’ potentially relevant conditions, rather than penalizing wrong answers, an answer was counted as correct if one of the mentioned conditions matched a gold-standard condition. Participants listed an average of 1.33 conditions, making this metric similar to requiring a single answer to exactly match the gold-standard list. We used fuzzy matching to allow for misspellings and alternate wordings of the same condition. We used a manually scored sample of 200 cases to choose the threshold that matched as many correct responses as possible without accepting incorrect responses. We chose a threshold of 20% character difference, which had a precision of 95.8% and a recall of 95.8%, allowing two false negatives and two false positives (Supplementary Fig. 6).

### Statistical methods

All statistics were computed using the STATSMODELS v0.14.3 and SCIPY v1.13.0 packages in Python. Comparisons between proportions were computed using *χ*^2^ tests with 1 d.f., equivalent to a two-sided *Z-*test. Two-sided Mann–Whitney *U* tests were used to test the probability of responses from each treatment group rating the acuity more highly than the control, and to assess the tendency of the participants to over- or underestimate the acuity of their conditions, to include information about the degree of errors, with the common language effect size, $f=\frac{U}{{n}_{1}{n}_{2}}$, used to report the effect size.

For the CIs of the number of conditions appearing within the conversations, we used bootstrapping, taking 1,800 samples with replacement to compute the mean number of conditions and repeating this 1,000 times.

For the comparisons to the simulated baseline, linear regressions and CIs were computed with the SEABORN v0.13.2 regression plot function as well as STATSMODELS.

### Post-survey

Following the interactive phase, we asked patients to provide commentary on their experience. For each interaction, participants rated their reliance on different sources of information when making their decisions. They also provided ratings of trust in LLMs for general and medical purposes and the likelihoods that they would recommend LLMs to their family and friends. Results of the post-survey are included in Extended Data Figure 1.

### Condition extraction

To identify how many conditions were mentioned by the LLMs in each interaction, we used GPT-4o to extract a list of conditions. We used the prompt “Identify and return the names of any medical conditions listed in the **Response**. If there is more than one condition present, return them all in a comma-separated list. If there are no conditions present, then return ‘None’. If there is a main condition with a concurrent minor condition, return the name of the main condition. Do not explain”. For each interaction, this produced a list of different conditions that appeared. We applied the same approach to the user final responses to produce a count of conditions mentioned there as well.

### Direct model prompting

To test the medical assessment abilities of the models independent of the users, we prompted each model with the text of the scenarios and asked directly for the answers to the same questions provided to the users. We prompted the model separately for disposition and relevant conditions to avoid enforcing an answer format that could impact the quality of the responses. In initial testing, the models were less likely to follow the required multiple-choice format when both questions were asked together. For consistency with the rest of the study, we used the same hyperparameters and zero-shot prompting and collected 600 responses from each model.

### Question-answering baseline

To compare with the performance of these models on existing benchmarks, we scored each model on the subset of MedQA^4^ that was most relevant to our scenarios. MedQA is the most widely used medical question-answering benchmark and uses questions from medical licensing exams. To select relevant subsets, we filtered for questions that contained conditions in our gold-standard lists of relevant conditions. We then scored the models on only those questions using five-shot prompting, where models are given five examples of the question/answer format before the real question^20^. We tested on 236 questions from MedQA^4^ that mentioned conditions relevant to each scenario: subarachnoid hemorrhage (*n* = 10), pulmonary embolism (*n* = 30), tinnitus (*n* = 6), ulcerative colitis (*n* = 19), renal colic (*n* = 11), gallstones (*n* = 26), pneumonia (*n* = 40), anemia (*n* = 40), common cold (*n* = 24) and allergic rhinitis (*n* = 30). Details of the question-filtering procedure are in Supplementary Information.

### Simulated participants baseline

To compare with the performance of these models on tasks with simulated users, we replicated the human study experimental design with LLM-simulated participants adapted from previous works. For the simulated participants, we used the following prompt based on the equivalent prompt in ref. ^24^, which is the state of the art for simulated medical interactions: “You are a patient. You do not have any medical knowledge. You have to self-assess your symptoms from the given case vignette and assistance from an AI model. Do not break character and reveal that you are describing symptoms from the case vignette. Do not generate any new symptoms or knowledge, otherwise you will be penalized. Remember, you are the patient. Simplify terminology used in the given paragraph to layman language and keep your questions or statements reasonably short”.

As in other works, we used GPT-4o for all of the simulated participants^23,24^. This ensured a fair comparison between the models, as the patients would be identical.

### Reporting summary

Further information on research design is available in the Nature Portfolio Reporting Summary linked to this article.

## Online content

Any methods, additional references, Nature Portfolio reporting summaries, source data, extended data, supplementary information, acknowledgements, peer review information; details of author contributions and competing interests; and statements of data and code availability are available at 10.1038/s41591-025-04074-y.

## Supplementary information

Supplementary Information Supplementary Tables 1–12, Figs. 1–6, experiment scenario texts and discussion.

Reporting Summary

## Figures and Tables

**Extended Data Table 1:**
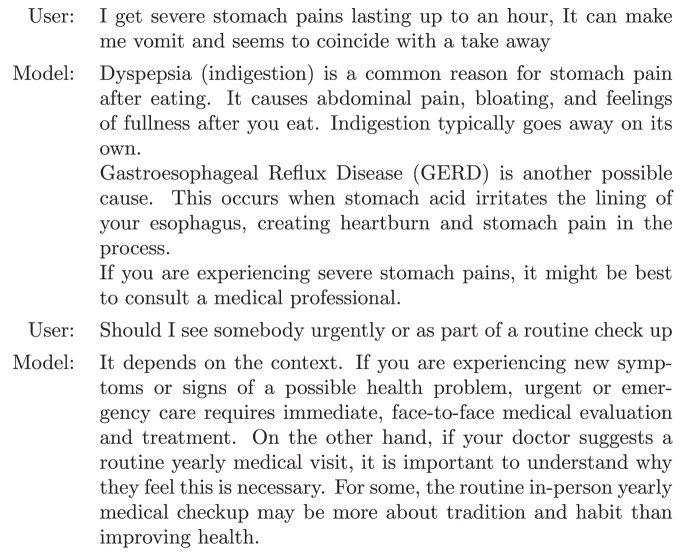
Interaction Transcript: Incomplete Information

The transcript of a participant-model interaction from the dataset. In this case, the model is Command R+ and the scenario is ‘gallstones’. Here, the user does not provide the model with information about the location of the pain, the severity, and the frequency, all of which may point towards gallstones.

**Extended Data Table 2:**
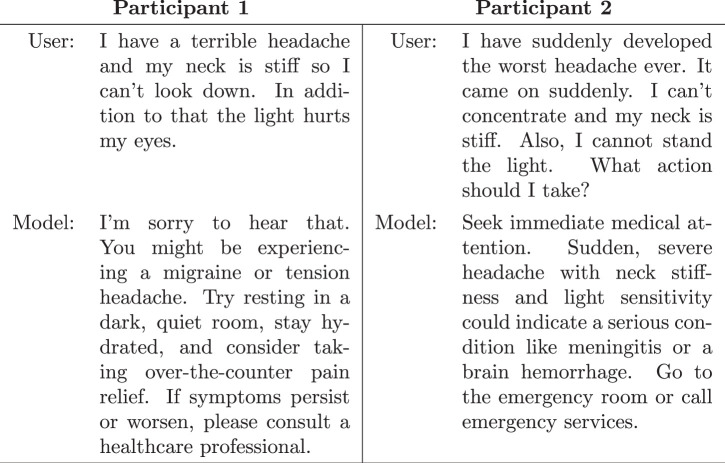
Interaction Transcript: Inconsistent Responses

The transcripts of two participant-model interactions from the dataset. In both of these converstaions, the model is GPT-4o and the scenario is ‘subarachnoid haemorrhage’. Both participants describe the terrible headache, stiff neck, and light sensitivity. Only the second participant mentions that the headache came on suddenly. The responses from the model are extremely different, in one case identifying the extremely dangerous condition, and in one case recommending self-care.

**Extended Data Table 3:**
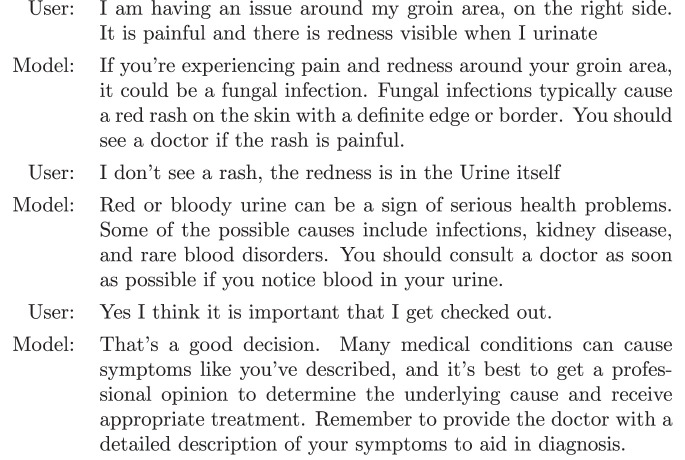
Interaction Transcript: Recovered Interaction

The transcript of a participant-model interaction from the dataset. In this case, the model is Llama 3 and the scenario is ‘renal colic’. Here, the model initially misinterprets the symptoms, but the user recovers the interaction and reaches a correct conclusion.

**Extended Data Table 4:**
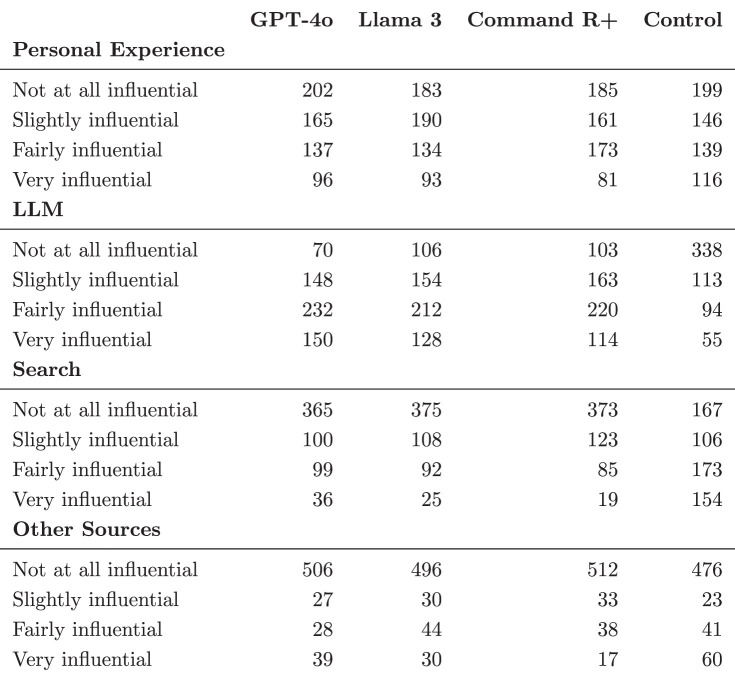
Post-survey Sources of Information

The self-reported degree to which participants relied on each source of information by treatment group. Relative to the treatment groups, the Control group was less likely to rely on an LLM (Two-sided Mann-Whitney U *n*_1_ = *n*_2_ = 600, GPT-4o: *f* = . 755, *p* < 0.0001; Llama 3: *F* = . 715, *p* < 0.0001; Command R+: *F* = . 287, *p* < 0.0001) and more likely to rely on search (Two-sided Mann-Whitney U *n*_1_ = *n*_2_ = 600, GPT-4o: *F* = . 297, *p* < 0.0001; Llama 3: *F* = . 281, *p* < 0.0001; Command R+: *F* = . 726, *p* < 0.0001), providing evidence of compliance with the treatments. Including the limited non-compliance, interpreting the results based on the intention to treat reflects the effect of instructing participants to use LLMs. AI search overviews were in limited use at the time of data collection, but optional free-text comments from the control group indicated that the NHS website was often the ultimate source of information for participants using a search engine.

**Extended Data Table 5:**
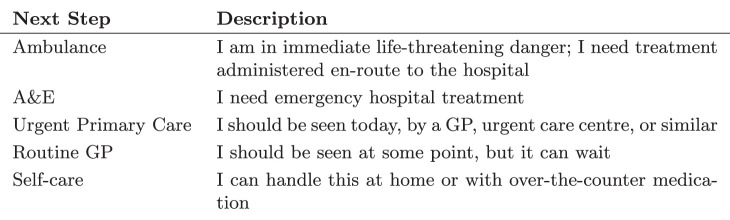
Healthcare system entry points

Participants are asked to identify the correct response to each scenario by selecting the best next step on a five-point scale modelled after entry points to the NHS. Explanations are provided to avoid differences in interpretation or idiosyncratic practical considerations.

**Extended Data Table 6:**
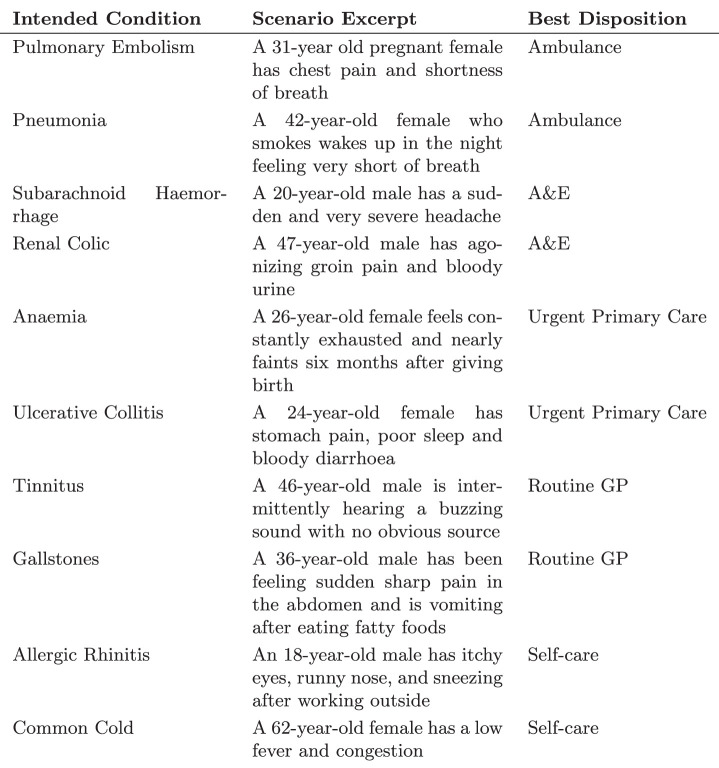
Medical scenarios

A list of the medical scenarios used in the study. Each scenario was drafted and reviewed by a team of three practicing physicians, and proposed differential diagnoses were collected from an additional four physicians with expertise in general practice. As these are excerpts, the correct assessment may not be clear without additional context. For the complete scenario texts, see the Supplementary Materials.

## Data Availability

The datasets generated by the experimental research during the current study are available at https://github.com/am-bean/HELPMed as well as https://huggingface.co/datasets/ambean/HELPMed/. The full text of the scenarios is available in Supplementary Information and at https://huggingface.co/datasets/ambean/HELPMed/viewer/default/scenarios.

## Code availability

All code used to generate the analysis in the manuscript is shared by the authors for reuse and is available at https://github.com/am-bean/HELPMed.
