# Publisher Correction: Reliability of LLMs as medical assistants for the general public: a randomized preregistered study

**DOI:** 10.1038/s41591-026-04404-8

**Published:** 2026-04-17

**Authors:** Andrew M. Bean, Rebecca Elizabeth Payne, Guy Parsons, Hannah Rose Kirk, Juan Ciro, Rafael Mosquera-Gómez, Sara Hincapié M, Aruna S. Ekanayaka, Lionel Tarassenko, Luc Rocher, Adam Mahdi

**Affiliations:** 1 Oxford Internet Institute, University of Oxford, Oxford, UK; 2 Nuffield Department of Primary Health Care Sciences, University of Oxford, Oxford, UK; 3 North Wales Medical School, Bangor University, Bangor, UK; 4 Betsi Cadwaladr University Health Board, Ysbyty Gwynedd, Bangor, UK; 5 National Health Service, London, UK; 6 Contextual AI, Mountain View, CA USA; 7 MLCommons, San Francisco, CA USA; 8 Factored AI, Palo Alto, CA USA; 9 Birmingham Women’s and Children’s NHS Foundation Trust, Birmingham, UK; 10 Institute of Biomedical Engineering, University of Oxford, Oxford, UK

**Keywords:** Social sciences, Health care

Correction to: *Nature Medicine* 10.1038/s41591-025-04074-y, published online 9 February 2026.

In the version of the article initially published, in Fig. 2a, the *y*-axis labels were “0, 20, 80, 60, 80, 100” in both graphs but should have been “0, 20, 40, 60, 80, 100”. This correction has been made to the HTML and PDF versions of the article.

